# Death in the Eyes of the Beholder

**Published:** 2010-05-01

**Authors:** S. Fry-Revere, B. Bastani

**Affiliations:** 1 *President, Center for Ethical Solutions, 40357 Featherbed Ln. Lovettsville, VA 20180, USA, *; 2 *Professor of Internal Medicine, Division of Nephrology, School of Medicine, St. Louis University, St. Louis, USA*

**Keywords:** Tissue and organ procurement, Death, Brain death, Electroencephalography, Organ transplantation

## Abstract

The US Uniform Determination of Death Act provides two alternatives for determining death—the circulatory criteria and the neurological criteria—yet history and the public’s current understanding of death in the US may mean that only brain death criteria can be relied upon without raising public suspicion that the medical profession is sacrificing the well-being of one group of patients (*i.e.*, those dying after traumatic injury) to save another group (*i.e.*, those in need of organs). The problem is exacerbated by existing debate on the appropriate waiting time after which death is inevitable and when the brain should be actually considered dead through prolonged absence of autoresuscitation. Given the difficulty of definitive determination of the time when brain function has ceased, two solutions are proposed: abandon the Dead Donor Rule or redefine death. Implementing the former would mean convincing the public to accept organ harvesting before the dying patient is completely brain dead through the writing of advance directives to permit organ harvest when death is inevitable though not confirmed. For the latter, reeducation would be necessary to persuade the public to accept the circulatory criteria for death as an independent determinant for death or the medical community would need to reconsider if the cessation of higher brain function is enough to be the basis for determining death. In conclusion, organ retrieval policies, no matter how medically sound, should seek to avoid the possibility of a public backlash that could result in fewer organs available for transplant.

The medical community in general, and the transplant medical community in particular, at least in the US, are suffering from an ever growing public mistrust. Every medical policy needs to keep this climate of doubt in mind. In a previous article, Dr. Delmonico argues forcefully and convincingly that there is widespread misunderstanding of the 1980 Uniform Determination of Death Act (UDDA), and while his medical analysis is sound, it could benefit from some contextual analysis.[1]

Delmonico points out that the UDDA sets out a definition of death with two alternative methods for determining when death occurs—circulatory-respiratory criteria and brain criteria. Delmonico further concluded that satisfaction of the circulatory-respiratory criteria for death necessarily means the brain criteria for death are also met without a need for further verification.[1] Our concern is not with these conclusions, but with their public ramifications. In a cultural milieu where medical professionals are viewed as barely more trustworthy than used car salesmen, organ retrieval policies, no matter how medically sound, should seek to avoid the possibility of a public backlash that could result in fewer organs available for transplant.

The atmosphere of mistrust of the medical establishment in the US has reached crisis proportions and cannot be ignored in the consideration of organ procurement policies. The problem is severe enough that the Harvard School of Public Health has a “Healthcare Trust Initiative” which recently published *The Trust Crisis in Healthcare: Causes, Consequences, and Cures *[2], and has several ongoing projects dealing with how to help physicians regain the trust of their patients and the general public.[3] Many of the general concerns about mistrust hit close to home. In a recent article, James DuBois who served on the Institutes of Medicine 2006 Committee on Increasing Rates of Organ Donation, discusses a finding that “25 percent or more of the members of groups surveyed expressed fears that if they signed a donor card, then physicians would do less to save their lives.” [4,5] One study published in 2006 in *Critical Care Medicine* shows that healthcare workers have concerns that the need for transplantable organs may cause a conflict of interest between the care given to dying patients and the care needed to preserve organs for transplantation.[6] Many articles and newspaper reports echo similar concerns about a tension between the organ shortage and the zeal exercised by organ procurement organizations in their attempts to increase donation.[7-9] Under these circumstances, extreme caution is needed to prevent the perception that physicians are establishing organ procurement policies to benefit certain patients at the expense of others and the general public.

Against this backdrop, we comment on Delmonico’s article [1]. First let us stress that we believe that Delmonico’s understanding of the UDDA is basically correct. The Act states in relevant part that “an individual who has sustained either [2] irreversible cessation of circulatory and respiratory functions, or [3] irreversible cessation of all functions of the entire brain, including the brainstem, is dead” [10]. Delmonico rightly observed that the definition is written in the alternative with one option being the use of brain criteria and the other option being the use of circulatory-respiratory criteria. However, when he argues that satisfaction of the first set of criteria (circulatory criteria) implies the inevitable satisfaction of the second set (brain criteria), Delmonico collapses the alternatives presented in the Act and creates a situation ripe for public misunderstanding.

Americans almost unanimously accept the second set of criteria (brain criteria) under the UDDA as criteria for determining death, but it seems that the first set of UDDA criteria, *i.e.*, irreversible loss of circulatory function, is less understood and mistrusted. [6,11] This is ironic since the irreversible cessation of brain function was introduced in 1968 by an *ad hoc* committee of the Harvard Medical School in order to supplement the older heart-lung criteria, not to replace them.[12] It seems that educating Americans to accept brain criteria for death has inadvertently conditioned the public not only to accept brain criteria as an alternative to circulatory criteria but to consider them as the only acceptable criteria.

The UDDA was written in 1980 to recognize both the older circulatory criteria and the newer brain criteria as alternative means of determining death.[13] Today, however, most people do not know that background or understand that “circulatory function” is a more medically concise means of identifying what used to be called “heart-lung” criteria. Unfortunately, understanding this history does little to help convince people to accept circulatory criteria as equally independently valid criteria for determining death. And as Delmonico’s article makes evident, even the transplant community is still debating whether both the UDDA alternatives for determining death are medically sound.

Delmonico argues that the two sets of criteria can be applied in the alternative, both being sufficient but neither necessary for determining death, yet he goes on to stress that satisfaction of circulatory criteria necessarily leads to the satisfaction of brain criteria. At first blush, considering cessation of circulatory function as an indicator of the cessation of brain function might be seen as a way to persuade the general public that circulatory criteria are just another way of determining brain death, but that is not what Delmonico says.[1] He writes “the consequence of the absence of circulation is upon the function of the brain. An irreversible loss of blood flow to the brain results in an irreversible loss of neurological function, the UDDA definition of death.”[1] The word “results” implies a causal relationship rather than a concomitant one. He quotes Bernat that “‘irreversible’ is an absolute and univocal condition that implies impossibility.” While it is understandable to stop treating someone who cannot be saved, it does not mean that it is appropriate to harvest that person’s organs before death occurs. Delmonico clearly agrees, but acknowledges that the period between the time when death is inevitable and when the brain is actually dead (as evidenced by a prolonged absence of autoresuscitation) is an empirical question. He describes how physicians disagree as to the appropriate waiting time, anywhere from 1.25 to 5 minutes. Such medical disagreements validate the public concern that organs are being harvested before people are actually dead. When Delmonico concedes that the value of circulatory criteria lies in their ability to foretell the inevitability of brain death, he validates the public perception that brain criteria, and not circulatory criteria, are ultimately the true determinant of death.

How should the transplant community react? The only effective way to assuage public concern would be to limit organ retrieval to patients for whom death has been confirmed in the standard manner of doing an electroencephalogram (EEG) to confirm brain death or by waiting long enough to be sure that there is no brain activity possible. Unfortunately, there are some reports indicating that brain stem activity may continue ten or more minutes after the absence of autoresuscitation, making it difficult, by some standards, to confirm that the patient is brain dead any sooner than 20 minutes after circulatory functions have stopped.[14,15] The statistical probability that all brain function will be lost five or six minutes after cessation of circulatory function is quite different from verifying that the entire brain has in fact stopped functioning.[16]

This discussion suggests two possible approaches for increasing the organ supply: abandon the Dead Donor Rule, or redefine death. Given the atmosphere of mistrust that permeates public perception of the organ procurement system, Delmonico quite rightly concluded that the Dead Donor Rule should be an immutable principle. But, if policymakers were to consider changing that rule, it would be essential to convince the public that it is acceptable to harvest organs before the dying patient is actually brain dead. Perhaps individuals could be given the option of writing advance directives that specifically permit their organs to be harvested if death is inevitable rather than only after death is confirmed.[17]

Alternatively, efforts could be made to persuade the public to accept definitions of death other than the whole brain death. Perhaps the public could be reeducated to accept circulatory criteria for death as an acceptable independent determinant for death as was the intention of the UDDA and as was the case before the 1968 Harvard *ad hoc* committee on brain death. If circulatory criteria are sufficient for a determination of death without any reference to brain criteria, then the only certainty required is that autoresuscitation is impossible. Or, the medical community could revisit the discussion of whether higher brain function criteria rather than the whole brain criteria should be the basis for determining death.[13,18] If such a change occurred, there would be no need to wait until a person loses his or her total brain function to harvest organs—loss of only higher brain function would be enough. But such a change would clearly entail a new definition of death, requiring amendments to both the UDDA and current medical standards of care. Without such significant shifts in understanding, retrieving organs without confirmation of death, as understood by the general public to mean loss of whole brain function, risks increasing public mistrust and consequently also risks of a decline in voluntary organ donation. 

In April 2009, the United Network for Organ Sharing reported a decline in voluntary organ donation for 2008. This was the first time in its 20-year history that there was a decline in donations and both living and cadaver donations were affected.[19] It is worth considering whether this new trend might be related to the public mistrust garnered by aggressive organ retrieval policies. It would behoove the transplant community to investigate this possibility and, in the meantime, proceed cautiously with the implementation of any organ retrieval policies that rely on anything other than whole brain criteria for the determination of death.
